# Immunophenotyping of Posttraumatic Neutrophils on a Routine Haematology Analyser

**DOI:** 10.1155/2012/509513

**Published:** 2012-02-21

**Authors:** Kathelijne Maaike Groeneveld, Marjolein Heeres, Loek Petrus Hendrikus Leenen, Albert Huisman, Leo Koenderman

**Affiliations:** ^1^Department of Trauma Surgery, University Medical Center Utrecht, 3508 GA Utrecht, The Netherlands; ^2^Department of Respiratory Medicine, University Medical Center Utrecht, 3508 GA Utrecht, The Netherlands; ^3^Department of Clinical Chemistry and Haematology, University Medical Center Utrecht, 3508 GA Utrecht, The Netherlands

## Abstract

*Introduction.* Flow cytometry markers have been proposed as useful predictors for the occurrence of posttraumatic inflammatory complications. However, currently the need for a dedicated laboratory and the labour-intensive analytical procedures make these markers less suitable for clinical practice. We tested an approach to overcome these limitations. *Material and Methods.* Neutrophils of healthy donors were incubated with antibodies commonly used in trauma research: CD11b (MAC-1), L-selectin (CD62L), Fc*γ*RIII (CD16), and Fc*γ*RII (CD32) in active form (MoPhab A27). Flow cytometric analysis was performed both on a FACSCalibur, a standard flow cytometer, and on a Cell-Dyn Sapphire, a routine haematology analyser. *Results.* There was a high level of agreement between the two types of analysers, with 41% for Fc*γ*RIII, 80% for L-selectin, 98% for CD11b, and even a 100% agreement for active Fc*γ*RII. Moreover, analysis on the routine haematology analyser was possible in less than a quarter of the time in comparison to the flow cytometer. *Conclusion.* Analysis of neutrophil phenotype on the Cell-Dyn Sapphire leads to the same conclusion compared to a standard flow cytometer. The markedly reduced time necessary for analysis and reduced labour intensity constitutes a step forward in implementation of this type of analysis in clinical diagnostics in trauma research.

## 1. Introduction

Trauma is a major cause of morbidity and mortality in people under the age of 50 in the western world [1]. This can be a direct result of the trauma and injury itself, or the postinjury immunological complications [2]. In response to tissue damage, due to trauma, an excessive immune reaction occurs. Dysfunctional polymorphonuclear (PMN) leukocytes play a clear role in the excessive immune response. This overwhelming immune response is considered to be a major risk factor in the development of posttraumatic organ failure [3, 4]. However, there remains a lack in the full understanding of the critical mechanisms and the identification of patients at risk for the development of systemic complication after trauma [5].

Inflammatory markers have been proposed as useful predictors for the occurrence of acute respiratory distress syndrome (ARDS) and multiple organ dysfunction syndrome (MODS) [6]. The development of immune status monitoring of trauma patients will not only help in the selection of patients at risk for posttraumatic complication but also may help in the choice of the most effective treatment protocol [7–9].

There are several markers indicated as possible indicators to predict the clinical course and clinical outcome of the trauma patient [10]. Our laboratory previously has shown that fMLF-induced active Fc*γ*RII (MhoPhab A27) can aid in early prediction which trauma patients are prone to develop inflammatory complications [10, 11]. This and other research on posttrauma immune responses depend on inflammatory markers measured by fluorescent monoclonal antibodies in a flow cytometer. However, experimental studies analysing inflammatory markers with flow cytometry need a dedicated laboratory and intensive laboratory work which is required for these analysis. This makes these inflammatory markers less attractive in clinical practice [5, 12]. Therefore, there is an indisputable need for a quick and easy-to-use, reliable, and cost-effective diagnostic analyser. So more research on the mediator cascade an diagnostic determination of trauma patients at risk can take place.

In most clinical laboratories of many hospitals worldwide, the routine analysis of blood is performed with an automated haematology analyser designed for counting peripheral blood cells in whole blood samples. One of these machines, the CELL-DYN Sapphire (CD-Sapphire), also offers fluorescent flow cytometry capacities. Compared to a conventional flow cytometer, the CD-Sapphire could provide a greater degree of automation and could thus be operated with relatively little training [13].

We, therefore, evaluated the performance of the CD-Sapphire in comparison to our routine flow cytometer for analysis of inflammatory markers.

## 2. Material and Methods

### 2.1. Reference Flow Cytometer

The reference flow cytometer used during this study was the FACSCalibur (Becton Dickinson, Mountain View, CA, USA). The FACSCalibur uses an argon gas laser at a fixed emission of 488 nm. The instrument is capable of detecting six parameters: forward scatter (FSC), side scatter (SSC), and three fluorescent emissions (FL-1: 530 ± 30 nm, FL-2:  585 ± 42 nm, and FL-3: >670 nm) utilizing the first laser. A smaller diode laser emitting red light at 635 nm is used to detect within the fourth fluorescent detector (FL-4: 661 ± 16 nm). Cells can be identified according to their specific forward- and side-scatter signals [14]. 

### 2.2. Cell-Dyn Sapphire

The CD-Sapphire (Abbott Diagnostics, Santa Clara, CA, USA) is a routine haematology analyser which uses spectrophotometry, electrical impedance, laser light scattering (multi angle polarized scatter separation, (MAPPS)), and 3 color fluorescent technologies to classify blood cells. There are detectors that are used for optical scatter (Axial Light Loss (ALL), 0°, cell size) and Intermediate Angle Scatter (IAS), 7°, cell complexity. Moreover, the analyser is equipped with an integrated fluorescence (488 blue diode) laser and three fluorescent detectors (FL-1: 530 ± 30 nm, FL-2: 580 ± 30 nm, and FL-3: 630 ± 30 nm). Fluorescence channels FL-1 and FL-2 are not routinely utilized. Therefore, they could be used for immunophenotyping [15]. The FL-3 fluorescence channel is used to quantify nucleated red blood cells and leukocyte viability with a propidium iodide staining. However, in a fresh sample, the large majority of cells is viable, and, therefore, also FL-3 could be used for the analysis of other fluorochromes.

The CD-Sapphire has a fully automated modus (CD3/4/8 modus) designed for sample preparation and determination of fluorescent characteristics of T-cell subsets, namely, CD3^+^CD4^+^ T-Helper and CD3^+^CD8^+^ T-suppressor cells [16]. The CD3/4/8 procedure uses three consecutive rack positions, where the first position is occupied by the patient sample and the second and third position are, respectively, occupied by the directly labelled CD3/CD4 and CD3/CD8 antibody mixtures. The analyser takes fixed volumes of blood from the first (patient) sample tube and injects this into the two reagent tubes. After mixing and a timed incubation period, aliquots of the blood antibody mixtures are aspirated and diluted prior to passage through the optical flow cell where measurements of the optical scatter and fluorescence are made. No sample washing is required, and red cell lysis is incorporated by the analyser in the automated procedure. The total time required for analysis and data acquisition is eight minutes.

The hardware and software configurations of the CD-Sapphire can be adapted to enable using this modus for alternative fluorescent reagents [15].

### 2.3. Sampling and Analysis

Blood (9 mL) of ten healthy adult donors was collected in sodium-heparine coated sterile tubes (Vacuette). Within one hour of sampling, blood samples were analysed for neutrophil phenotype with the use of flow cytometry. The analysis of neutrophil receptor expression profiles was described previously [11]. In short, the samples were cooled on melting ice and kept on ice during the whole procedure. Directly labeled monoclonal antibodies were added to whole blood according the recommendations of the manufacturer and incubated for 60 minutes on ice. We chose four antibodies that are known for their significant importance in research in trauma patients: RPE-labeled CD11b (clone 2LPM19c) from Dako, Glostrup, Denmark, Alexa Fluor 647-labeled CD16 (clone 3G8), from Scientific Group, BD Pharmingen, Milnerton for the experiment on the CD-Sapphire and ECD-labeled CD16 (clone 3G8) for the experiment on the FACSCalibur, from Beckman Coulter, Marseille, France, FITC-labeled CD62L (clone Dreg56) from BD Pharmingen, USA, and FITC-labeled A27, a monoclonal phage antibody, which recognizes active Fc*γ*RII (active CD32), was manufactured in the Department of Respiratory Medicine at the University Medical Centre Utrecht (clone MoPhab A27) [17].

After incubation, the red cells were lysed with ice-cold isotonic NH_4_Cl. After a final wash with PBS2+ phosphate-buffered saline with added sodium citrate (0.38% wt/vol) and pasteurized plasma proteins (10% vol/vol), each of the samples were both analysed on the reference flow cytometer and on the CD-Sapphire.

To test the applicability of the fully automated CD3/4/8 modus, whole blood samples of ten healthy volunteers were stimulated at 37°C for 5 minutes with N-formyl-methionyl-leucyl-phenylaline (fMLF 10^-6 ^mol/L). After stimulation, the samples were kept on room temperature until analysis with the CD3/4/8 modus. As described previously [15, 18], the two reaction tubes normally containing CD3/CD4 antibodies were replaced by two barcoded nonanticoagulate Vacutainer tubes, containing FITC-labeled A27, the antibodies directed against active Fc*γ*RII.

### 2.4. Data Processing

On completion of analysis on the CD-Sapphire and FACSCalibur, raw data files were downloaded and transferred to a PC for manual software analysis (FCS Express Version 3; De Novo Software, Thornhill, Ontario, Canada) of fluorescence.

### 2.5. Statistical Analysis

Data from individual experiments are depicted as median fluorescence intensity (MFI) in arbitrary units (AUs) and standard deviation of at least 5.000 events. Results in figures are presented as means ± standard error of means (SEMs), unless otherwise specified. To test the association between results from the two flow cytometers, a Spearman correlation coefficient was defined. Statistical analysis of paired measurements was performed with the nonparametric Wilcoxon signed rang test. Statistical significance was defined as *P* < 0.05. All data was analysed using SPSS version 17.0 (SPSS Inc., Chicago, IL, USA) and GraphPad Prism (GraphPad Software Inc., La Jolla, CA, USA).

## 3. Results

**Figure 1:**
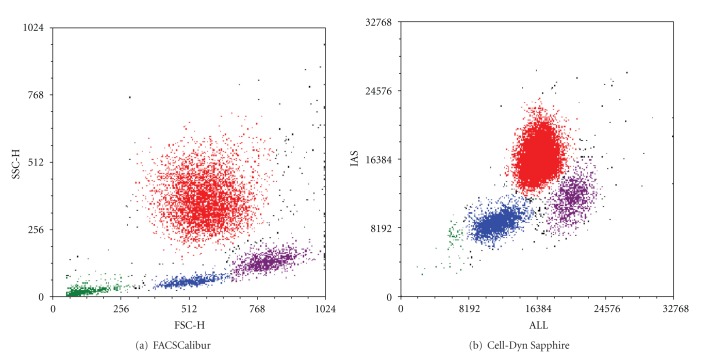
Morphological display of leukocytes. Representative example of the morphological plots of a blood sample from a healthy volunteer on (a) a FACSCalibur and (b) a Cell-Dyn Sapphire. The cells in red are the neutrophils, the purple population are the monocytes, the blue cells are lymphocytes, and the puple in green is debris. (a) Forward Scatter (FSC)—Side Scatter (SSC) display from a FACSCalibur. FSC represents the size/cell volume of cells, SSC represents the inner complexity of the particle. (b) Multi Angle Polarised Scatter Separation (MAPSS) leukocyte differential plot from a Cell-Dyn Sapphire. Axial Light Loss (ALL) is an indicator of cell size, Intermediate Angle Scatter (IAS) represents the cell complexity.

Representative images of the gating strategies for individual leukocyte populations can be seen in Figure 1. These data show that the scatter plots for size (ALL/FSC) and complexity (IAS/SSC) produced by both analysers are very similar. This allows a comparable gating strategy in both analysers.

**Figure 2:**
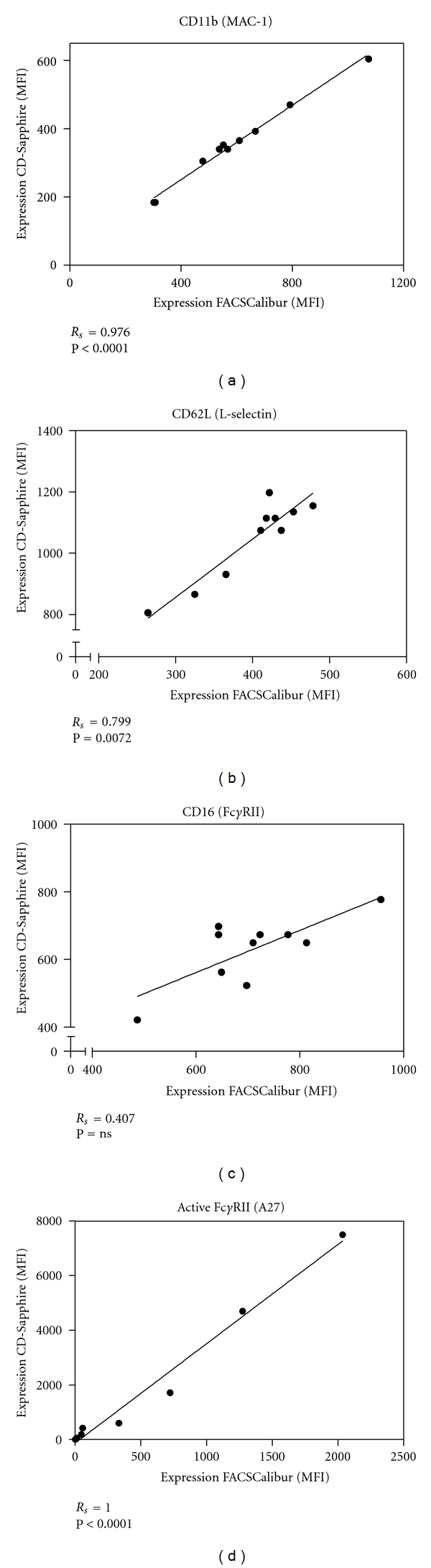
Agreement between two flow cytometers. Samples of whole blood of ten healthy volunteers were measured on both a FACSCalibur and a Cell-Dyn Sapphire to demonstrate the agreement between median fluorescent intensity (MFI) of monoclonal antibodies on neutrophils. Whole blood was incubated with (a) RPE-labeled CD11b (MAC-1); (b) FITC-labeled CD62L (L-selectin); (c) Alexa Fluor 647-labeled CD16 (Fc*γ*RIII) for the experiment on the CD-Sapphire and ECD-labeled CD16 (Fc*γ*RIII) for the experiment on the FACSCalibur; (d) FITC-labeled MhoPhab A27, a monoclonal phage antibody, which recognizes active Fc*γ*RII (CD32). The solid lines are regression lines. *R*_*s*_ = Spearmans rank correlation coefficient. ns = Significant.

The agreement between the measurements on the routine flow cytometer and the CD-Sapphire was good for all markers (Figure 2). For MAC-1, the Spearman correlation coefficient was 0.976 (*P* < 0.001), L-selectin had a coefficient of 0.799 (*P* = 0.0072), Fc*γ*RIII of 0.407 (*P* = ns), and for active Fc*γ*RII the association was 1.000 (*P* < 0.0001).

The average time for preparation and analysis of one sample on the routine flow cytometer was approximately 125 minutes, whereas sample preparation and analysis on the CD-Sapphire took approximately 130 minutes. Calculations of absolute fluorescence/cell values took about 10 minutes in both machines.

**Figure 3:**
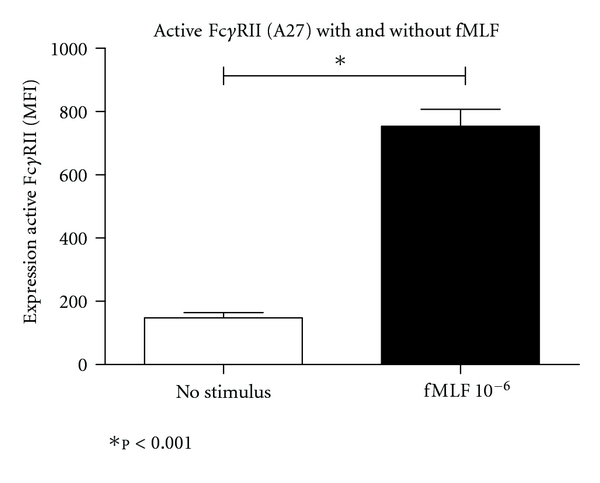
Incubation of monoclonal antibodies by the Cell-Dyn Sapphire. In ten healthy volunteers, two blood samples were taken. Half of the whole blood samples (*n* = 10) were preincubated for 5 minutes with fMLF 10^−6^, and half of samples (*n* = 10) were not preincubated. The samples were then put in a Cell-Dyn Sapphire, and this analyser fully automatic performed flow cytometry analysis with the monoclonal phage antibody A27 that recognizes the active Fc*γ*RII (represented as expression of active Fc*γ*RII). MFI = median fluorescence intensity.

In the second set of experiments, we used a fully automated staining procedure by the CD-Sapphire, including pipetting, lysing of erythrocytes and measuring fluorescence (Figure 3). This approach resulted in the identification of significant differences in expression of active Fc*γ*RII, between samples without preincubation with fMLF (mean 148.1 ± 53.0), and samples with fMLF preincubation (mean 753.9 ± 168.5, *P* < 0.001). The average time for the whole procedure, including sample preparation and analysis of one sample on the CD-Sapphire, took approximately 18 minutes. Manual off-line calculations of absolute fluorescence/cell values took about 10 minutes.

## 4. Discussion

This study evaluated the implementation of monoclonal antibodies for the determination of the posttraumatic systemic immune responses on a routine haematology analyser. The CD-Sapphire is such an analyser with a capability for analysis of fluorescent cells, by applying methods that are analogous to conventional flow cytometry. This study demonstrates the feasibility of analysis of the activation phenotype of neutrophils by using the extended immuno-fluorescent modus of the CD-Sapphire analyser. This was shown for inflammatory markers that are commonly used in trauma research.

The results from the CD-Sapphire showed a high agreement to those of a routine flow cytometer, when paired samples were analysed. These results are in line with studies from the field of clinical chemistry, showing that results from the CD-Sapphire were comparable to their conventional flow cytometry in diagnostic measurements [13, 15, 18]. In addition, the machine has a fully automated modus for staining and incubating whole blood samples with antibodies, lysis of erythrocytes, and subsequent data acquisition [14]. Using this modus, we were able to show that the haematology analyser could clearly distinguish neutrophils that were activated with fMLF compared to control cells. This automated analysis only required a fraction of the time needed for regular flow cytometric analyses. In addition, this analysis did not require sample preparation by technicians. Implementation of such screening on activation markers on neutrophils of trauma patients with the CD-Sapphire greatly facilitates research on the posttraumatic immune response and its modulation. Furthermore, the rapid and fully automated modus does not need specialized staff trained in flow cytometry and is likely cost effective.

There are some limitations to these experiments. Firstly, the FACSCalibur and the CD-Sapphire have slightly different fluorescent detectors. We, therefore, could not use the same fluorochrome in our CD16 antibody (clone 3G8) preparations.

Secondly, we measured the expression of Fc*γ*RIII in the FL-3 channel, normally used to test the viability of cells with Propidium Iodine. We used fresh blood samples (<1 hour of sampling), assuming that the large majority of cells had a full viability. Müller et al. and others previously showed that proportions of nonviable leukocytes (especially neutrophils) progressively increase between twelve and 72 hours of storage at room temperature [19]. In comparison, Hedberg and Lehto found that white blood cell differential parameters are stable for up to 48–72 hours, when stored at >4°C [20]. We, therefore, advise that analysis of additional fluorescent markers in FL-3 of the CD-Sapphire should be performed without delay after sampling of the whole blood.

Thirdly, CD-Sapphire software includes fully automated data analysis of fluorescent measurements in the CD3/4/8 modus for lymphocytes. However, to obtain absolute fluorescent information of other cell populations, currently measured raw file information must be extracted from the machine and subjected to manual data analysis in off-line flow analysis software, such as FCS Express. These manual interventions could be overcome by the development of automated software, such as the Leuko64 QuantiCALC software that already exists for the analysis the LeukoCD64 Assay kits [21].

## 5. Conclusion

In conclusion, this study shows that the CD-Sapphire haematology analyser can be used for analysis of inflammatory antibodies to determine the posttraumatic immune response in trauma patients. It is, not only, comparable with conventional flow cytometry, but it is also more suitable for personnel without specific flow cytometry training. The reduced time necessary for analysis and potentially reduced labour cost constitutes a step forward in further research possibilities and for implementation in clinical diagnostics.
